# Complete Mitochondrial Genome Sequence and Phylogenetic Analysis of *Procambarus clarkii* and *Cambaroides dauricus* from China

**DOI:** 10.3390/ijms241411282

**Published:** 2023-07-10

**Authors:** Liang Luo, Yue Xu, Shihui Wang, Rui Zhang, Kun Guo, Wei Xu, Zhigang Zhao

**Affiliations:** 1 Key Open Laboratory of Cold Water Fish Germplasm Resources and Breeding of Heilongjiang Province, Heilongjiang River Fishery Research Institute, Chinese Academy of Fishery Sciences, Harbin 150070, China; luoliang@hrfri.ac.cn (L.L.); wangshihui@hrfri.ac.cn (S.W.); zhangrui@hrfri.ac.cn (R.Z.); guokun@hrfri.ac.cn (K.G.); xuwei@hrfri.ac.cn (W.X.); 2 Interdisciplinary Centre of Marine and Environmental Research, University of Porto, 4450-208 Porto, Portugal; a74445@ualg.pt

**Keywords:** *Procambarus clarkii*, *Cambaroides dauricus*, mitogenome, phylogenetic relationship

## Abstract

To enhance the management and protection of crayfish genetic diversity and germplasm resources in *Cambaroides dauricus* (*C. dauricus*), a common species of *Procambarus clarkii* (*P. clarkii*) was used as a control group to compare the whole mitochondrial genome sequence using Illumina sequencing technology. This study found that the mitochondrial genome of *C. dauricus* is 15580 bp in length, with a base composition of A (31.84%), G (17.66%), C (9.42%), and T (41.08%) and a C + G content of 27.08%. The C + G in the D-loop is rich in 17.06%, indicating a significant preference. The mitochondrial genome of *C. dauricus* contains 13 protein-coding genes, 22 tRNA genes, and 2 rRNA genes, with most of the genes labeled in the negative direction, except for a few genes that are labeled in the positive direction. The start codons of the ten coding sequences are ATG, and the quintessential TAA and TAG are the stop codons. This study also found that the Ka/Ks ratios of most protein-coding genes in the mitochondria of both shrimps are lower than 1, indicating weak natural selection, except for nad 2, nad 5, and cox 1. The Ka/Ks ratio of cox 3 is the lowest (less than 0.1), indicating that this protein-coding gene bears strong natural selection pressure and functional constraint in the process of mitochondrial genetic evolution of both shrimps. Furthermore, we constructed phylogenetic analyses based on the entire sequence, which effectively distinguishes the high body from other shrimp species of the genus based on the mitochondrial genome. This study provides molecular genetic data for the diversity investigation and protection of fishery resources with Chinese characteristics and a scientific reference for the evolutionary study of Procambarus.

## 1. Introduction

Although *Cambaroides dauricus* (*C. dauricus*) is only found in the Heilongjiang, Jilin, and Liaoning provinces of Northeast China, it has potential for freshwater cultivation due to its desirable characteristics such as flavorful meat, high nutrient content, rapid fertility, dietary versatility, and continuous improvement pace [1]. Meanwhile, *Procambarus clarkii* (*P. clarkii*) is the most commonly cultivated freshwater crayfish species worldwide, especially in the middle and lower reaches of the Yangtze River in China [2,3]. A temperature of 25 °C is ideal for its growth and development, and *P. clarkii* has become a significant source of high-quality protein [4] and a valuable economic resource. However, while there have been many studies on the reproductive habits [5,6,7,8] and larval nutrition [9,10] of wild *P. clarkii*, the artificial propagation technique is still immature, resulting in overfishing and depletion of juvenile resources [11].

Fortunately, *C. dauricus*, a close relative of *P. clarkii*, has been actively researched for its fundamental ecology and aquaculture technologies, and is more tolerant to cold temperatures, with an optimal water temperature range of 16–21 °C [12,13,14]. Additionally, research on both species in agriculture has increased, although the mitochondrial genomes of these two shrimp species have not been extensively studied in comparison to other shrimp species. Therefore, this study aims to combine molecular genetics to clarify population genetic diversity, breed connections, and the phylogenetic process of variation generation in both species to better understand Astacidae genotypes.

As is widely known, cross-species genetic diversity is determined by changes in heterozygotes and variables that impact gene frequency, including migration, mutation, selection, and genetic drift [15]. Population genetic analysis, on the other hand, seeks to describe the extent of interactive visualization of genetic differences and to explain the variability in genetic mutations [15]. For aquaculture, mitochondrial genome DNA (mtDNA) is purely maternally inherited and self-replicating [16,17]. As a result, mtDNA has been broadly utilized in the domains of phylogeny, population genetics, and adaptive evolution in fish [18,19,20,21], as well as being used more rarely in the studies of shrimp. Because it has been demonstrated that using longer mitochondrial genome sequences to examine evolutionary connections across species is far more reliable [22,23], no relevant research with comparative study of the entire sequences of the mitochondrial genomes of adjacent shrimp species is available. Thus, the fundamental properties and sequencing discrepancies of the mitochondrial genomes of shrimp from two distinct cultivars are compared in this study; factors including mitogenomic organizations, gene rearrangements, nucleotide compositions, protein-coding genes (PCG)codon usages, secondary structures of transfer genes (tRNA), and control regions (CRs)were examined in *C. dauricus* and *P. clarkii*, which were rebuilt to assess the validity of the newly available molecular data for the evolutionary connections of Astacidae further.

## 2. Results

### 2.1. Structural Characteristics of the Mitochondrial Genomes

**Figure 1:**
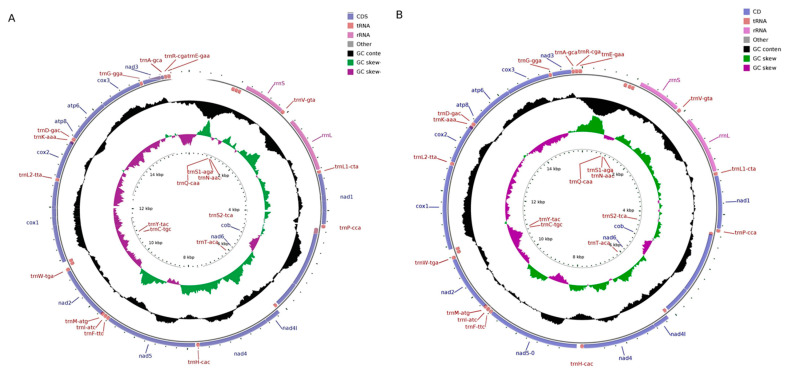
Mitochondrial maps of *P. clarkii* (**A**) and *C. dauricus* (**B**). The mitochondrial maps of *P. clarkii* (**A**) and *C. dauricus* (**B**) depict the genes transcribed clockwise outside the map, and counterclockwise inside the map. The inner circles show the deviation of GC content and GC skew from the average value of the entire sequence.

In this study, we annotated and submitted the completed mitochondrial genome sequences of *P. clarkii* (accession no: OL542520) and *C. dauricus* (accession no: OL542521) to GenBank. The mitogenomes of both species were typical double-stranded circular molecules, with lengths of 15,937 bp (Figure 1A) for *P. clarkii* and 15,580 bp (Figure 1B) for *C. dauricus*, and they displayed similar mitogenome maps. The base composition of *P. clarkii* was A (31.84%), G (17.66%), C (9.42%), and T (41.08%), with a significantly richer content of C + G (27.08%) than A + T (72.92%), indicating a preference for CG. Similarly, *C. dauricus* also exhibited a CG preference, with a higher proportion of C + G (27.50%) than A + T (72.50%).

The mitochondrial DNA structure of *P. clarkia* and *C. dauricus* is consistent with that of other crayfish, comprising 37 genes in the standard set, including 13 protein-coding genes (*cox 1-3*, *ATP6*, *ATP8*, *ND1-6*, *ND4L*, and *Cytb*), 22 tRNA genes, 2 rRNA genes, and a control region (D-loop region). These genes are listed in Table 1 and Table 2 for *P. clarkii* and *C. dauricus*, respectively. In *P. clarkii*, four pairs of adjoining genes had 9 overlapping nucleotides, while in *C. dauricus*, nine gene limitations had 36 overlapping nucleotides. There were 227 intergenic nucleotides (IGNs) at 20 locations in *P. clarkii* and 478 IGNs at 19 locations in *C. dauricus*, suggesting a loose structure of the mitochondrial DNA. The entire mitochondrial genomes of both species were heavily skewed towards A and T nucleic acids with 72.92% and 72.50%, respectively, with *P. clarkii* having a negative AT skew and a positive GC skew, while *C. dauricus* had a positive AT skew and a negative GC skew.

### 2.2. Structural Analysis of rRNA, tRNA, and D-Loop Regions

Table 1 and Table 2 reveal that both *P. clarkii* and *C. dauricus* have *rrnS* and *rrnL* in their mitochondrial genome. In *P. clarkii*, *rrnS* is 793 bp long and is located between *trnN*-aac and *trnV*-gta, while *rrnL* is 1050 bp long and is located between *trnV*-gta and *trnL1*-cta. Similarly, in *C. dauricus*, *rrnS* is 791 bp long and is positioned between *trnN*-aac and *trnV*-gta, while *rrnL* is 1054 bp long and is placed between *trnV*-gta and *trnL1*-cta. *rrnL* is more conserved than *rrnS* in both species.

The length of the 22 tRNAs in *P. clarkii* is 1422 bp, ranging from 61 to 68 bp, with *trnA*-gca being the shortest and *trnQ*-caa being the longest. Fifteen tRNAs are labeled in the negative direction, and *trnQ*-caa, *trnS1*-aga, *trnN*-aac, *trnS2*-tca, *trnT*-aca, *trnC*-tgc, and *trnY*-tac are marked in the positive direction.

Similarly, in *C. dauricus*, the total length of the 22 tRNAs is 1480 bp, ranging from 61 to 69 bp, with *trnA*-gca and *trnF*-ttc being the shortest and *trnQ*-caa being the longest. Fifteen tRNAs are also labeled in the negative direction, and *trnQ*-caa, *trnS1*-aga, *trnN*-aac, *trnT*-aca, *trnS2*-tca, *trnC*-tgc, and *trnY*-tac are marked in the positive direction.

### 2.3. Predicted Structures of rRNA, tRNA, and D-Loop Regions

**Figure 2:**
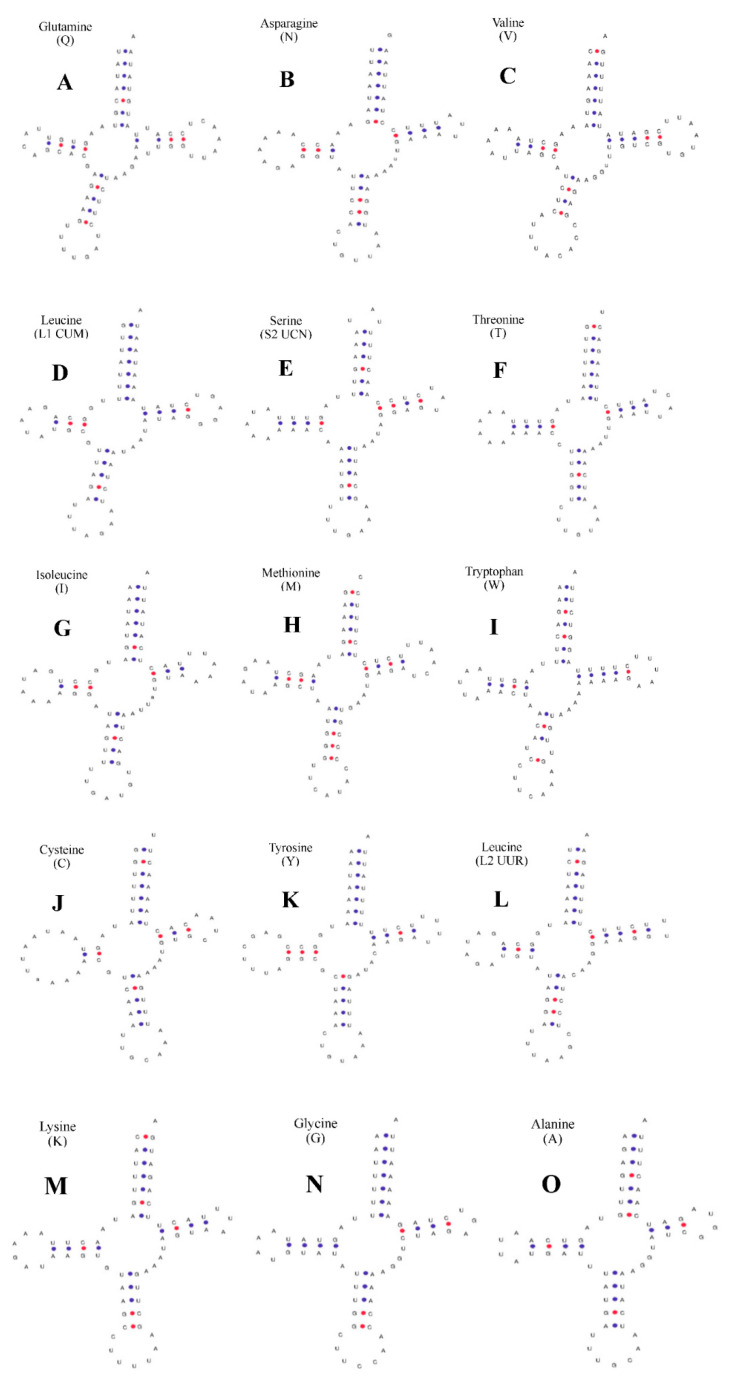
Secondary structure of tRNA gene in mitochondrial genome of *P. clarkii.* (**A**) *trnQ* (Glutamine). (**B**) *trnN* (Asparagine). (**C**) *trnV* (Valine). (**D**) *trnL1* (CUN) (Leucine). (**E**) *trnS2* (UCN) (Serine). (**F**) *trnT* (Threonine). (**G**) *trnI* (Isoleucine). (**H**) *trnM* (Methionine). (**I**) *trnW* (Tryptophan). (**J**) *trnC* (Cystine). (**K**) *trnY* (Tyrosine). (**L**) *trnL2* (UUR) (Leucine). (**M**) *trnK* (Lysine). (**N**) *trnG* (Glycine). (**O**) *trnA* (Alanine). The tRNA genes are labelled with their corresponding amino acids. The blue dot represent A-U pairs; The red dot represent C-G pairs.

For *P. clarkii* (Figure 2), 6 tRNAs, namely *trnS1*-aga, *trnP*-cca, *trnH*-cac, *trnF*-ttc, *trnD*-gac, and *trnE*-gaa, lacked the dihydrouracil arm (DHU arm), while the other 15 tRNAs contained typical “clover” secondary structures that could be predicted online using tRNAscan-SE. The C-T conversion in the amino acid arm caused U-U mismatches in *trnQ*-caa (Figure 2A) and *trnW*-tga (Figure 2I), resulting in the deletion of the DHU ring in *trnL2*-tta (Figure 2L). The control region (D-loop) of *P. clarkii* is located between the *trnE*-gaa and *trnQ*-caa genes, with a total length of 844 bp, and no tandem repeats were found using the tandem repeat finder software. The base content and percentage were as follows: A 42.54%, T 40.40%, C 4.86%, G 12.20%. A + T were rich in 82.94% and C + G were rich in 17.06%, which showed a clear preference for C + G and an anti-adenine phenomenon.

**Figure 3:**
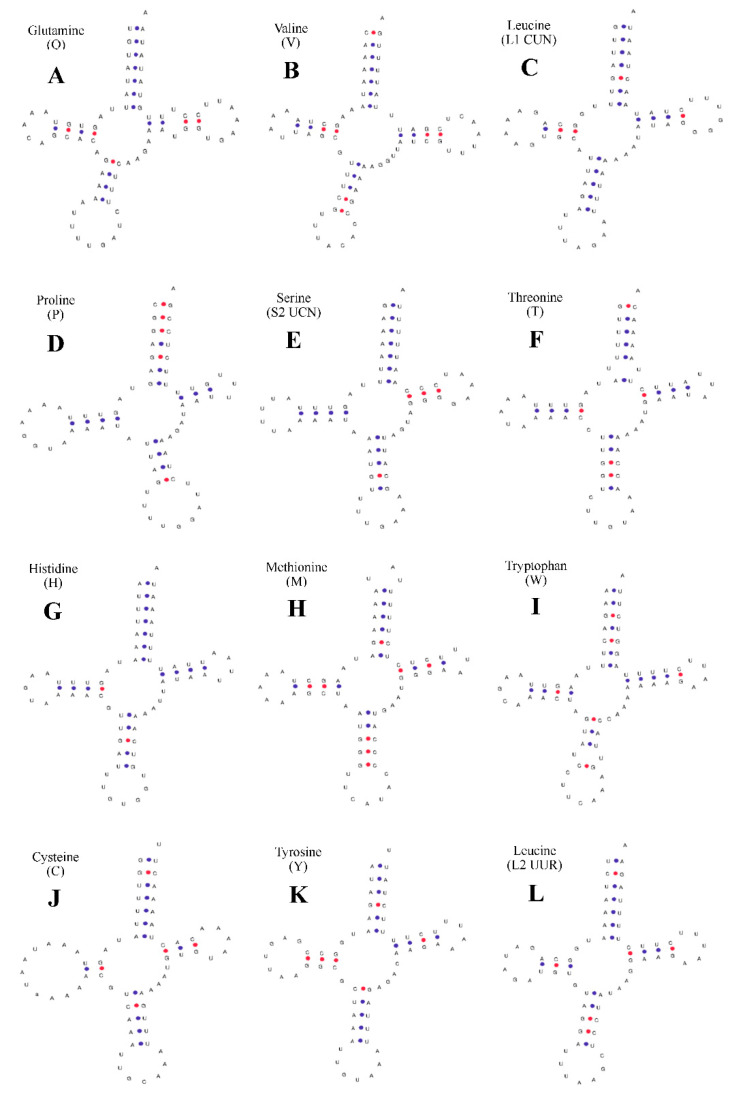

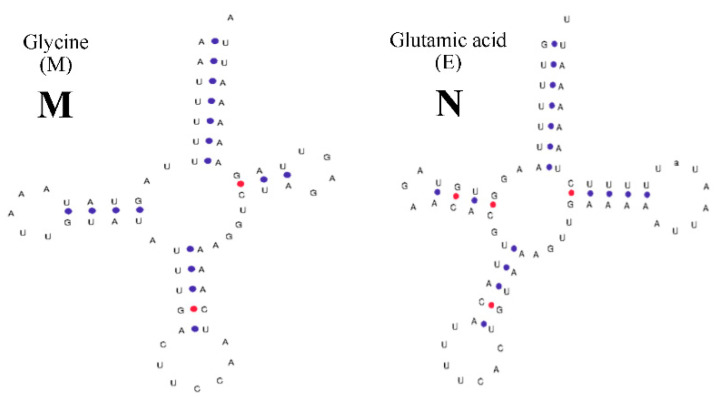
Secondary structure of tRNA gene in mitochondrial genome of *C. dauricus.* (**A**) *trnQ* (Glutamine). (**B**) *trnV* (Valine). (**C**) *trnL1* (CUN) (Leucine). (**D**) *trnP* (Proline). (**E**) *trnS2* (UCN) (Serine). (**F**) *trnT* (Threonine). (**G**) *trnH* (Histidine). (**H**) *trnM* (Methionine). (**I**) *trnW* (Tryptophan). (**J**) *trnC* (Cystine). (**K**) *trnY* (Tyrosine). **(L**) *trnL2* (UUR) (Leucine). (**M**) *trnM* (Methionine). (**N**) *trnE* (Glutamic acid). The tRNA genes are labelled with their corresponding amino acids. The blue dot represent A-U pairs; The red dot represent C-G pairs.

For *C. dauricus* (Figure 3), the DHU arm was missing in *trnS1*-aga, *trnN*-aac, *trnF*-ttc, *trnI*-atc, *trnK*-aaa, *trnD*-gac, *trnG*-gga, *trnA*-gca, and *trnR*-cga, while the other 13 tRNAs contained typical “clover” secondary structures, which were predicted online using tRNAscan-SE. The C-T conversion in these peptide arms caused a U-U mismatch in *trnQ*-caa (Figure 3A) and *trnW*-tga (Figure 3I). The control region (D-loop) of *C. dauricus* is located between the *trnE*-gaa and *trnQ*-caa genes, with a total length of 791 bp, and no tandem repeats were found using the software Tandem Repeat Finder. The base content and percentage were as follows: A 43.87%, T 40.58%, C 3.79%, G 11.76%. A + T was rich in 84.45%, while C + G was rich in 15.55%, indicating a clear preference for C + G and an anti-adenine phenomenon.

### 2.4. Analysis of the Codon Preference Profiles in Protein-Encoding Genes

In this study, we separately analyzed the codons of protein-encoding genes in *P. clarkii* and *C. dauricus* using frequency and relative synonymous codon usage (RSCU), as presented in Table 3 and Table 4. For *P. clarkii* (Table 3), a total of 3750 codons were encoded, with the bold font indicating the codon with the most usage for the same amino acid. RSCU values were greater than 1 for all codons, except AUG (M), UGG (W), and CGA (R), which means that the remaining codons were used with preference. Furthermore, the UNN-type codons accounted for 35.92% of the total number of codons. With the exception of UGG (W, 0.69), the RSCU of other UNN-type codons was greater than 1, indicating that the second and third codon sites were G and thus used more frequently. For C. dauricus (Table 4), a total of 3733 codons were encoded, and the bold font indicates the codon with the most usage for the same amino acid. RSCU values were greater than 1 for all codons, except AGU (S), UGG (W), and CGA (R), indicating preference for the remaining codons. Furthermore, the UNN-type codons accounted for 35.59% of the total number of codons. With the exception of UGG (W, 0.85), the RSCU of other UNN-type codons was greater than 1, indicating that the second and third codon sites were G and thus used more frequently.

### 2.5. The Genetic Variation of the Mitochondrial Genome

PAML was used to calculate the non-synonymous substitution rate (Ka) and synonymous substitution rate (Ks) of protein-coding genes in the mitochondrial genomes of *P. clarkii* and *C. dauricus*. The results showed that with the exceptions of nad2, nad5, and cox1, the Ka/Ks ratio of the remaining 13 protein-coding genes was less than 1 (ranging from 0.0582 to 0.7266), indicating that these genes were subjected to specific negative purification selection during the evolutionary processes of coding sequences in both species, as shown in Table 5.

The Ka/Ks ratios of the nad5 (2.2423) and nad2 (1.3190) genes were found to be greater than 1, indicating that these genes were subject to positive or productive natural selection. The Ka and Ks values of the nad2 gene were similar, suggesting that this gene underwent mostly neutral selection with inadequate natural selection pressure. However, the difference between the Ka and Ks values was much larger for the nad5 gene, resulting in a higher Ka/Ks ratio and demonstrating that biological evolution was a strong driving force in the evolutionary process of this gene.

The cox1 gene had a Ka/Ks ratio of 2.1049, indicating that it underwent certain selection pressure during evolution. The Ka/Ks ratio of the cox3 gene was the lowest among all genes studied, with a value of less than 0.1. This suggests that protein-coding genes were under strong natural selection pressure, bound by the protein function encoded by the gene, to ensure the normal biological function of the protein encoded by the cox3 gene. Thus, the cox3 gene plays an important role in the survival and evolution of both *P. clarkii* and *C. dauricus*.

### 2.6. The Phylogenetic Analysis

**Figure 4:**
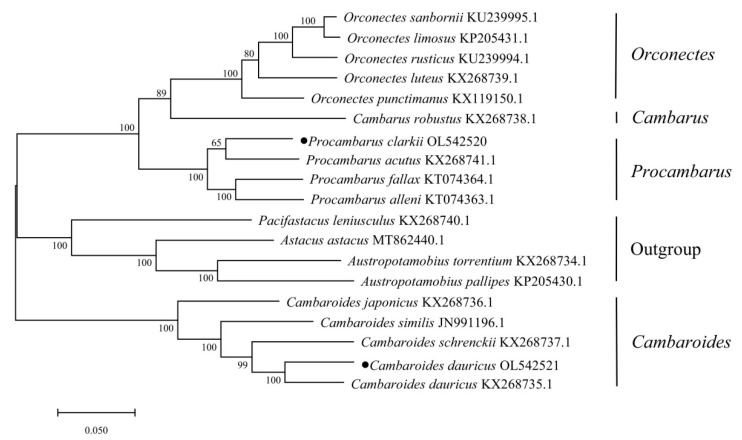
Phylogenetic relationships constructed using maximum likelihood method based on mitochondrial genome.

The evolutionary model was used to construct a phylogenetic tree (Figure 4) based on the nucleotide sequences of 13 protein-coding genes from 18 mitochondrial genomes. The results indicate that each shrimp species has its own branch, and the shrimps of *Orconectes*, *Procambraus Cambaroides*, and *Austropotamobius* first gather into a single fine branch. Then, the three species of *Pacifastacus leniusculus*, *Astacus astacus*, and *Austropotamobius* form a small branch of parallel evolution, forming an independent evolutionary branch with *Cambaroides*. Additionally, *Procambraus* and *Orconectes* join into a single evolutionary branch. These results are consistent with the taxonomic kinship of *Astacidae*, which is in line with the traditional classification based on morphological identification.

## 3. Discussion

In this study, the complete mitochondrial genome sequence of *P. clarkii* and *C. dauricus* was determined, with a total length of 15,937 bp and 15,580 bp. The base composition of *P. clarkii* was A (42.54%), T (40.40%), C (4.86%), and G (12.20%), and the composition ratio of *C. dauricus* was A (43.87%), T (40.58%), C (3.79%), and G (11.76%). The above situation follows an apparent A + T preference; the C + G content was about 17.06% and 15.55%, respectively, far less than the A + T content (82.94%, 84.45%). The mitochondrial base structure of vertebrates is similar [24,25]. Among them, the high guanine (G) content in the body of the fish is similar to that of other bony fish such as *Seriola lalandi* (17.84%) [26], *Sillago aeolus* (18.75%) [27], and *Oryzias celebensis* (17.60%) [28], exhibiting significant resistance to bird uric acid. This study systematically examined the two mitochondrial genomes’ genomic structural characteristics, base composition, codon preference, and protein-coding genes. The results showed that 13 proteins were similar in their mitochondrial genomes, showing significant anti cytosine phenomena in *P. clarkii* and *C. dauricus*.

We found that the overlap length of the two animals was between 1 and 46 bp. The largest overlap fragment was between the protein-coding genes *cox2* and *trnLys(k)* (46 bp), while the overlap fragment between *ATP8* and *ATP6* was 6 bp, the overlap fragment between *cox3* and *trnGly(G)* was 1 bp, and the overlap fragment between *trnSer(UCN)* and *CYTB* was 16 bp for *P. clarkii* and 19 bp for *C. dauricus*. The overlap fragment between *trnMet(M)* and *ND2* was 23 bp for *P. clarkii* and 20 bp for *C. dauricus*, while the overlap fragment between *trnTyr(Y)* and *cox1* was 7 bp for *P. clarkii* and 2 bp for *C. dauricus*. In addition, *P. clarkii* had a 1 bp overlap fragment between *ND2* and *trnTrp(W)*, and *ND3* and *trnAla(A)*. In fish, the overlap fragment is generally only 7–10 bp [29], while in mammals, it is generally 40–46 bp [30].

The start codons for *P. clarkii* were ATG or ATT, and the stop codons were TAA or TAG in this study, and the start codons for *C. dauricus* were ATG, ATT, or ATT, and the stop codons were TAA or TAG. Both species showed positive and negative chain specificity. The codon preference analysis showed that the frequency of NNU-type codons was the highest, which was consistent with the preference of the base T in their protein-coding gene composition, and similar to the results of crustaceans [31].

The 22 tRNA genes in the mitochondrial genomes of the two animal species show no variation. In *P. clarkii*, 15 tRNA genes have typical cloverleaf structures, while *trnS1*-aga, *trnP*-cca, *trnH*-cac, *trnF*-ttc, *trnD*-gac, and *trnE*-gaa lack the dihydrouridine arm (DHU stem) and form a simple loop at the corresponding position. In *C. dauricus*, 13 tRNA genes have typical cloverleaf structures, while *trnS1*-aga, *trnN*-aac, *trnF*-ttc, *trnI*-atc, *trk*-aaa, *trnD*-gac, *trnG*-gga, *trnA*-gca, and *trnR*-cga lack the dihydrouridine arm and form a simple loop at the corresponding position. The secondary structures of different tRNAs may differ due to the effects of missing aminoacyl arms, unstable structures, and non-typical base pairing, which may be caused by external environmental factors [32].

The Ka/Ks ratio can be used to determine the degree of selective pressure on protein-coding genes. Ka/Ks > 1 indicates positive selection, Ka/Ks = 1 indicates neutral evolution, and Ka/Ks < 1 indicates purifying selection [33,34]. The Ka/Ks values of nad5, nad2, and cox1 are greater than 1, indicating that these genes are affected by positive or productive natural selection, while the other genes have values less than 1, indicating that they are affected by purifying selection. The Ka/Ks value is generally considered the best parameter to represent the selective pressure on protein-coding genes.

According to the above data, we know that *Cambarus* phylogenetic trees were constructed partly through the maximum likelihood method based on their complete mitochondrial genome sequence. *Orconectes*, *Procambraus Cambaroides*, and *Austropotamobius* create a single acceptable subsidiary, followed by *Pacifastacus leniusculus, Astacus astacus*, and *Austropotamobius* forming one tiny strand of parallel evolution to form an independent evolutionary branch with the *Cambaroides*. This is consistent with the classification results obtained by Zheng Wenjuan et al. using a single gene, 16S rRNA [35,36].

## 4. Materials and Methods

### 4.1. Sample Collection and Identification and DNA Extraction

*P. clarkii* were collected at the Yangtze River Fisheries Research Institute of Chinese Academy of Fishery Sciences while *C. dauricus* were selected at Qingdingzi forest farm (Huinan County, Tonghua City, China). The morphological traits of these specimens were promptly recognized by the Heilongjiang River Fishery Research Institute of Chinese Academy of Fishery Sciences, and then they were preserved in 95% ethanol and kept at −80 °C. Total DNA was isolated from muscle tissues according to the manufacturer’s instructions using an E.Z.N.A.^®^ Tissue DNA Kit (Omega, Norcross, GA, USA).

The proposed DNA concentration and purity were determined subsequently utilizing a NanoDrop 8000 Spectrophotometer (NanoDrop Technologies, Wilmington, NC, USA). In this study, the genomic DNA of *P. clarkii* and *C. dauricus* was amplified through PCR using a total of 25 L, including template DNA 1 μL, 2 × Taq PCR Master Mix 12.5 μL, 1 μL each upper and downstream primers (10 mol·L^−1^),and dd H_2_O 9.5 μL. Amplification reactions were performed according to the following procedure: 94 °C 5 min; 94 °C 30 s, 56 °C 30 s, 72 °C 30 s (total 30 cycles); 72 °C 5 min; kept at 4 °C. All PCR products were detected using 1% agarose gels containing ethidium bromide, purified with the AxyPrep DNA Gel Extraction Kit (Axygen Biosciences, Union City, CA, USA), and quantified using QuantiFluor™-ST (Promega, Madison, WI, USA). Bidirectional sequencing was performed by BIOZERON Co., Ltd., Shanghai, China.

### 4.2. Sequence Assembly

Total genomic DNA was extracted using a modified cetyltrimethylammonium bromide (CTAB) method and 500-bp paired-end library construction was applied using the NEBNext Ultra DNA Library Prep Kit for Illumina sequencing. Sequencing was carried out on the Illumina NovaSeq 6000 platform (BIOZERON Co., Ltd., Shanghai, China). Approximately 2 Gb of raw data from *Cambaroides dauricus* (GenBank No. OL542521) was generated, with 150 bp paired-end read lengths. For PacBio library construction and sequencing, more than 5 μg of sheared and concentrated DNA was subjected to size selection using the Blue Pippin system (Sage Science, Beverly, MA, USA). Approximately 20 kb SMRTbell libraries were prepared according to the manufacturer’s instructions (PacBio, Menlo Park, CA, USA). One SMRT cell and 4.5 Gb data composed of 35.9 million reads were sequenced on the PacBio Sequel platform.

De novo assembly with GetOrganelle v1.6.4 referencing the mt genome of closely related species *Procambarus clarkii*-ref (GenBank No. OL542520) produced contigs of the mt genome. A number of potential mitochondrion reads were extracted from the pool of Illumina reads using BLAST searches against the mt genomes of related species *Procambarus clarkii*-ref and the GetOrganelle result. The mitochondrion Illumina reads were obtained to perform cp genome de novo assembly using the SPAdes-3.13.1 package. Clean PacBio long reads were aligned against the GetOrganelle and SPAdes assembled scaffolds using the BLASR program. All aligned PacBio reads were extracted to perform self-correction and mt genome de novo assembly using the Canu v2.1.1 package, followed by error correction using Pilon v1.22. The GetOrganelle assembly contigs were optimized using the scaffolds from SPAdes-3.13.0 result. Finally, the assembled sequences were reordered and oriented according to the reference mt genome, thus generating the final assembled mitochondrion genomic sequence.

### 4.3. Gene Annotation

The mitochondrion genes were annotated using the online MITOS tool, using default parameters to predict protein-coding genes, transfer RNA (tRNA) genes, and ribosome RNA (rRNA) genes [37]. The position of each coding gene was determined using BLAST searches against ref mt genes. Manual corrections of genes for start/stop codons were performed in SnapGene Viewer by referencing the ref mt genome. The circular mt genome map of *P. clarkii* and *C. dauricus* was drawn using the CGview tool (http://stothard.afns.ualberta.ca/cgview_server/, accessed on 19 May 2023). Functional annotations were performed using sequence similarity Blast searches with a typical cut-off E-value of 10^−5^ against several publicly available protein databases: the NCBI non-redundant (Nr) protein database, Swiss-Prot, Clusters of Orthologous Groups (COGs), and Kyoto Encyclopedia of Genes and Genomes (KEGG) and Gene Ontology (GO) terms.

### 4.4. Sequence Feature Analysis

The nucleotide makeup and codon usage of mitochondrial genome sequences of *P. clarkii* and *C. dauricus* were estimated using MEGA [38], while calculations for AT and GC offset were used [39]. Then, DNA SP 5.0 [40] was used to examine gene features and variation locations for the principal mRNA transcripts.

### 4.5. The Phylogenetic Analysis of Genetic Variation in the Mitochondrial Genome

Mega 7.0 software was used to perform the preference and phylogenetic analysis on the high body base composition and codons. Table 6 shows the mitochondrial genome sequences of 18 scad species obtained from the GenBank. A maximum likelihood evolutionary tree was constructed after finishing multiple sequence alignments and 1000 bootstrap tests.

## 5. Conclusions

This study suggests that *P. clarkii* and *C. dauricus* may belong to the same population, as indicated by their similar mitochondrial genome structure. However, there may be some degree of geographic isolation between the two species. Despite this, *P. clarkii* and *C. dauricus* were effectively differentiated from other crayfish genera by utilizing both morphological and bioinformatic approaches. These findings can contribute to the development of techniques for species identification, population division, and the preservation and sustainable use of germplasm resources. Ultimately, this research can help promote the sustainable and healthy growth of the aquaculture industry in China and worldwide.

## Figures and Tables

**Table 1:** Mitochondrial genome structure of *P. clarkii*.

Gene	Position (bp)	Size (bp)	Direction	Intergenic Nucleotides	Anti- or Start/ Stop Codons	A + T%
*D-loop*	1–844	844		−	-	82.9
*trnGln(Q)*	845–912	68	+	−	-	69.1
*trnSer1(AGN)*	914–980	67	+	1	-	71.6
*trnAsn(N)*	981–1044	64	+	0	-	75.0
*rrnS*	1111–1903	793	−	66	-	75.4
*trnVal (V)*	1903–1972	70	−	−1	-	68.6
*rrnL*	2174–3223	1050	−	201	-	74.8
*trnLeu1(CUN)*	3238–3300	63	−	14	-	74.6
*ND1*	3304–4266	963	−	3	ATG/ATG	71.5
*trnPro (P)*	4275–4339	65	−	8	-	76.9
*trnSer2(UCN)*	4342–4404	63	+	2	-	77.8
*CYTB*	4388–5539	1152	+	−17	ATG/TAA	68.3
*ND6*	5539–6051	513	+	−1	ATT/TAA	72.1
*trnThr(T)*	6073–6137	65	+	21	-	78.5
*ND4L*	6140–6433	294	−	2	ATG/TAA	75.9
*ND4*	6433–7776	1344	−	−1	ATG/TAA	71.9
*trnHis(H)*	7776–7840	65	−	−1	-	84.6
*ND5*	7859–9574	1716	−	18	ATG/TAA	73.4
*trnPhe(F)*	9574–9636	63	−	−1	-	73.0
*trnIle(I)*	9637–9699	63	−	0	-	76.2
*trnMet(M)*	9701–9767	67	−	1	-	61.2
*ND2*	9744–10,760	1017	−	−24	ATG/TAA	74.5
*trnTrp(W)*	10,759–10,825	67	−	−2	-	77.6
*trnCys(C)*	10,827–10,890	64	+	1	-	76.6
*trnTyr(Y)*	10,891–10,955	65	+	0	-	72.3
*COX1*	10,948–12,492	1545	−	−8	ATT/TAA	68.4
*trnL2 (UUR)*	12,494–12,556	63	−	1	-	66.7
*COX2*	12,557–13,291	735	−	0	ATG/TAG	71.2
*trnLys(K)*	13,245–13,308	64	−	−47	-	87.5
*trnAsp(D)*	13,311–13,377	67	−	2	-	86.6
*ATP8*	13,378–13,536	159	−	0	ATG/TAA	75.5
*ATP6*	13,530–14,204	675	−	−7	ATG/TAA	72.9
*COX3*	14,204–14,992	789	−	−1	ATG/TAA	67.0
*trnGly(G)*	14,991–15,052	62	−	−2	-	74.2
*ND3*	15,059–15,406	348	−	6	ATT/TAG	69.5
*trnAla(A)*	15,405–15,465	61	−	−2	-	70.5
*trnArg(R)*	15,468–15,529	62	−	2	-	67.7
*trnGlu(E)*	15,530–15,593	64	−	0	-	71.9

**Table 2:** Mitochondrial genome structure of *C. dauricus*.

Gene	Position (bp)	Size (bp)	Direction	Intergenic Nucleotides	Anti- or Start/Stop Codons	A + T%
*D-loop*	1–791	791		−	−	84.5
*trnGln(Q)*	792–860	69	+	−	−	73.9
*trnSer1(AGN)*	877–943	67	+	16	−	67.2
*trnAsn(N)*	944–1007	64	+	0	−	75.0
*rrnS*	1084–1874	791	−	76	−	75.7
*trnVal (V)*	1897–1964	68	−	22	−	70.6
*rrnL*	2166–3219	1054	−	201	−	74.6
*trnLeu1(CUN)*	3233–3297	65	−	13	−	72.3
*ND1*	3301–4263	963	−	3	ATG/TAG	72.5
*trnPro (P)*	4271–4334	64	−	7	−	71.9
*trnSer2(UCN)*	4338–4401	64	+	3	−	78.1
*CYTB*	4382–5536	1155	+	−20	ATG/TAA	68.8
*ND6*	5536–6048	513	+	−1	ATT/TAA	75.0
*trnThr(T)*	6072–6134	63	+	23	−	79.4
*ND4L*	6137–6430	294	−	2	ATG/TAA	74.1
*ND4*	6430–7770	1341	−	−1	ATG/TAA	73.6
*trnHis(H)*	7770–7833	64	−	−1	−	84.4
*ND5*	7894–9564	1671	−	60	−	71.9
*trnPhe(F)*	9564–9624	61	−	−1	−	72.1
*trnIle(I)*	9628–9691	64	−	3	−	73.4
*trnMet(M)*	9695–9757	63	−	3	−	68.3
*ND2*	9737–10,750	1014	−	−1	ATG/TAA	83.3
*trnTrp(W)*	10,750–10,815	66	−	−1	−	72.7
*trnCys(C)*	10,815–10,879	65	+	−1	−	78.5
*trnTyr(Y)*	10,880–10,942	63	+	0	−	68.3
*COX1*	10,940–12,478	1539	−	−3	ATC/TAG	66.8
*trnL2 (UUR)*	12,481–12,544	64	−	2	−	68.8
*COX2*	12,545–13,276	732	−	0	ATG/TAG	68.0
*trnLys(K)*	13,230–13,293	64	−	−47	−	75.0
*trnAsp(D)*	13,295–13,358	64	−	1	−	87.5
*ATP8*	13,359–13,517	159	−	0	ATG/TAA	75.5
*ATP6*	13,511–14,185	675	−	−7	ATG/TAA	69.8
*COX3*	14,185–14,973	789	−	−1	ATG/TAA	66.5
*trnGly(G)*	14,972–15,033	62	−	−2	−	79.0
*ND3*	15,040–15,387	348	−	6	ATT/TAA	70.7
*trnAla(A)*	15,389–15,449	61	−	1	−	68.9
*trnArg(R)*	15,450–15,512	63	−	0	−	68.3
*trnGlu(E)*	15,513–15,580	68	−	0	−	77.9

**Table 3:** Codon preference statistics of 13 protein-coding genes of *P. clarkii*.

Codon	Count	RSCU	Codon	Count	RSCU	Codon	Count	RSCU	Codon	Count	RSCU
**UUU (F)**	299	1.84	**UCU (S)**	140	2.89	**UAU (Y)**	144	1.88	**UGU (C)**	40	1.69
UUC (F)	27	0.16	UCC (S)	9	0.40	UAC (Y)	12	0.12	UGC (C)	0	0.00
**UUA (L)**	419	4.29	UCA (S)	58	1.20	UAA (*)	10	0.46	UGA (*)	79	2.38
UUG (L)	76	0.81	UCG (S)	6	0.09	UAG (*)	3	0.16	**UGG (W)**	25	0.69
CUU (L)	45	0.55	**CCU (P)**	97	2.82	**CAU (H)**	65	1.25	CGU (R)	23	0.70
CUC (L)	6	0.05	CCC (P)	12	0.32	CAC (H)	12	0.44	CGC (R)	2	0.06
CUA (L)	25	0.27	CCA (P)	22	0.48	**CAA (Q)**	40	1.10	**CGA (R)**	26	0.92
CUG (L)	4	0.04	CCG (P)	3	0.07	CAG (Q)	24	0.59	CGG (R)	8	0.68
**AUU (I)**	302	1.85	**ACU (T)**	101	2.47	**AAU (N)**	117	1.75	**AGU (S)**	62	1.33
AUC (I)	11	0.07	ACC (T)	14	0.22	AAC (N)	21	0.25	AGC (S)	5	0.10
AUA (I)	179	1.08	ACA (T)	42	0.85	**AAA (K)**	61	1.43	**AGA (R)**	65	2.13
**AUG (M)**	50	0.85	ACG (T)	8	0.15	AAG (K)	30	0.57	AGG (R)	53	1.51
GUU (V)	104	1.28	**GCU (A)**	106	2.43	**GAU (D)**	71	1.77	**GGU (G)**	86	1.27
GUC (V)	11	0.19	GCC (A)	18	0.59	GAC (D)	6	0.23	GGC (G)	15	0.22
**GUA (V)**	131	1.97	GCA (A)	35	0.78	**GAA (E)**	46	1.11	GGA (G)	70	1.40
GUG (V)	47	0.56	GCG (A)	10	0.19	GAG (E)	40	0.89	GGG (G)	72	1.10

Note: The bold fonts in the table are amino acid preference codons; The letter in parentheses represent the abbreviations of each amino acid name; * the termination codon.

**Table 4:** Codon preference statistics of 13 protein-coding genes of *C. dauricus*.

Codon	Count	RSCU	Codon	Count	RSCU	Codon	Count	RSCU	Codon	Count	RSCU
**UUU (F)**	306	1.82	**UCU (S)**	140	2.81	**UAU (Y)**	121	1.60	**UGU (C)**	36	1.40
UUC (F)	29	0.18	UCC (S)	22	0.47	UAC (Y)	30	0.40	UGC (C)	5	0.13
**UUA (L)**	394	4.01	UCA (S)	64	1.69	UAA (*)	10	0.67	UGA (*)	69	2.20
UUG (L)	58	0.59	UCG (S)	9	0.18	UAG (*)	3	0.13	**UGG (W)**	32	0.85
CUU (L)	83	0.82	**CCU (P)**	74	2.25	**CAU (H)**	65	1.32	CGU (R)	16	0.45
CUC (L)	10	0.08	CCC (P)	15	0.45	CAC (H)	13	0.37	CGC (R)	2	0.12
CUA (L)	44	0.45	CCA (P)	34	0.75	**CAA (Q)**	51	1.23	**CGA (R)**	28	0.89
CUG (L)	6	0.05	CCG (P)	7	0.25	CAG (Q)	16	0.62	CGG (R)	13	0.45
**AUU (I)**	283	1.86	**ACU (T)**	91	2.28	**AAU (N)**	121	1.48	**AGU (S)**	34	0.72
AUC (I)	24	0.15	ACC (T)	14	0.24	AAC (N)	27	0.52	AGC (S)	9	0.14
AUA (I)	182	0.99	ACA (T)	46	1.01	**AAA (K)**	65	1.36	**AGA (R)**	90	2.80
**AUG (M)**	28	1.00	ACG (T)	9	0.15	AAG (K)	25	0.64	AGG (R)	41	1.29
**GUU (V)**	124	1.81	**GCU (A)**	101	2.13	**GAU (D)**	45	1.28	GGU (G)	52	1.13
GUC (V)	10	0.18	GCC (A)	10	0.32	GAC (D)	21	0.41	GGC (G)	17	0.27
GUA (V)	106	1.68	GCA (A)	60	1.40	**GAA (E)**	49	1.20	**GGA (G)**	111	1.57
GUG (V)	22	0.32	GCG (A)	10	0.16	GAG (E)	35	0.80	GGG (G)	64	1.03

Note: The bold fonts in the table are amino acid preference codons; The letter in parentheses represent the abbreviations of each amino acid name; * the termination codon.

**Table 5:** Analysis of mitochondrial gene coding rate.

Gene	Total Number of Sites	Non-Synonymous Substitution Rate (Ka)	Synonymous Substitution Rate (Ks)	Ka/Ks Ratio
*nad1*	963	0.1640	0.7698	0.2130
*cob*	1152	0.1204	1.3004	0.0926
*nad6*	513	0.9050	1.0257	0.8823
*nad4l*	294	0.1541	0.7320	0.2105
*nad4*	1341	0.7720	0.8137	0.9488
*nad5*	1671	1.9679	0.8773	2.2431
*nad2*	1014	1.5361	1.1646	1.3190
*cox1*	1539	2.2137	1.0517	2.1049
*cox2*	732	0.5639	1.3191	0.4275
*atp8*	159	0.3522	0.8095	0.4351
*atp6*	675	0.1645	0.6540	0.2515
*cox3*	789	0.0758	0.8173	0.0927
*nad3*	348	0.2033	1.4072	0.1445

**Table 6:** The origins of mitochondrial genomes.

Family	Subfamily	Genus	Species	Accession Number
*Astacidae*	*Cambarinas*	*Cambaroides*	*Cambaroides dauricus*	OL542521
			*Cambaroides dauricus*	KX268735.1
			*Cambaroides similis*	JN991196.1
			*Cambaroides japonicas*	KX268736.1
			*Cambaroides schrenckii*	KX268737.1
		*Cambarus*	*Cambarus robustus*	KX268738.1
		*Orconectes*	*Orconectes punctimanus*	KX119150.1
			*Orconectes luteus*	KX268739.1
			*Orconectes rusticus*	KU239994.1
			*Orconectes limosus*	KP205431.1
			*Orconectes sanbornii*	KU239995.1
		*pacifastacus*	*pacifastacus leniusculus*	KX268740.1
		*procambarus*	*Procambarus clarkii*	OL542520
			*procambarus alleni*	KT074363.1
			*Procambarus fallax*	KT074364.1
			*Procambarus acutus*	KX268741.1
		*Astacus*	*Astacus astacus*	MT862440.1
		*Austropotamobius*	*Austropotamobius torrentium*	KX268734.1
			*Austropotamobius pallipes*	KP205430.1

## Data Availability

The complete mitogenome sequence with gene annotation has been submitted to the NCBI GenBank under the accession numbers OL542520 and OL542521. The sequence data utilized in this study can be found in the Supplementary Materials (S1–S4).

## Supplementary Materials

The supporting information can be downloaded at: https://www.mdpi.com/article/10.3390/ijms241411282/s1.
